# Sleeping and waking-state oral behaviors in TMD patients: their correlates with jaw functional limitation and psychological distress

**DOI:** 10.1007/s00784-024-05730-2

**Published:** 2024-05-22

**Authors:** Adrian Ujin Yap, Sunghae Kim, Byeong-min Lee, Jung Hwan Jo, Ji Woon Park

**Affiliations:** 1 Department of Dentistry, Ng Teng Fong General Hospital and Faculty of Dentistry, National University Health System, Singapore, Singapore; 2 National Dental Research Institute Singapore, National Dental Centre Singapore and Duke-NUS Medical School, Singapore Health Services, Singapore, Singapore; 3 Dental Research Institute, Seoul National University, Seoul, Korea; 4 Center for Future Dentistry, Seoul National University School of Dentistry, Seoul, Korea; 5 Department of Oral Medicine, Seoul National University Dental Hospital, Seoul, Korea; 6 Department of Oral Medicine & Oral Diagnosis, Seoul National University School of Dentistry, 101 Daehak-Ro, Jongno-Gu, Seoul, Korea

**Keywords:** Temporomandibular disorders, Diagnosis, Oral behaviors, Physiology, Psychological distress

## Abstract

**Objectives:**

This study investigated oral behaviors in various temporomandibular disorder (TMD) subtypes, assessing their frequency, extent, and associations with both jaw functional status and psychological distress.

**Materials and methods:**

Anonymized data from consecutive "initial-visit" TMD patients at a university-affiliated oral medicine clinic were obtained. Alongside demographic information, patients completed various questionnaires including the Diagnostic Criteria for TMD (DC/TMD) Symptom Questionnaire, Oral Behavior Checklist (OBC), Jaw Functional Limitation Scale-20 (JFLS-20), Patient Health Questionnaire-9 (PHQ-9), and General Anxiety Disorder Scale-7 (GAD-7). Patients underwent a protocolized clinical examination and received diagnoses of pain-related (PT), intra-articular (IT), or combined (CT) TMD using the DC/TMD diagnostic algorithms. Data were evaluated with Chi-square/non-parametric tests and logistic regression analyses (α = 0.05).

**Results:**

The study comprised 700 patients (mean age 37.4 ± 15.7 years), with 12.6%, 15.1%, and 72.3% diagnosed with PT, IT, and CT, respectively. For all TMD subtypes, oral activities during sleep were more prevalent than those during wakefulness. While variations in total/subscale OBC scores were insignificant, substantial differences were observed in global/subscale JFLS (PT, CT > IT), depression (PT, CT > IT), and anxiety (CT > IT) scores. Near-moderate correlations (*r*_*s*_ = 0,36–0.39) were discerned between overall/waking-state non-functional oral behaviors and depression/anxiety. Multivariate analysis indicated that the odds of different TMD subtypes were influenced by sex, age, and jaw functional status.

**Conclusions:**

For all TMD patients, sleep-related oral activities were more commonly reported than waking-state activities. Factors such as sex, age, and jaw functional limitation are associated with the likelihood of different TMD subtypes.

**Statement of clinical relevance:**

Oral behaviors, in themselves, do not predict distinct TMD subtypes, in contrast to factors such as sex, age, and jaw functional status.

## Introduction

Temporomandibular disorders (TMD), comprising a cluster of clinical conditions affecting the masticatory system, pose a significant public health problem, impacting as much as 16% of the adult population [1–3]. They are the second most common musculoskeletal issue causing chronic pain and disability, following low back pain [4]. Females, especially those in their reproductive years, have been found to have an elevated susceptibility to TMD [1, 3]. TMDs may exhibit overlapping symptoms with other chronic pain disorders, such as fibromyalgia and primary headaches, which could be attributed to the phenomenon of central sensitization [5]. In accordance with the Diagnostic Criteria for TMD (DC/TMD) and its tiered reporting structure, TMD can be categorized into pain-related (primarily myalgia, arthralgia, and headache attributed to TMD), intra-articular (primarily disc displacement, degenerative joint disease, Temporomandibular joint [TMJ] subluxation), and combined conditions [2, 6].

The complex etiology of TMD is influenced by a combination of biological and psychosocial factors, encompassing genetics, sex hormones, poor general health, somatic symptoms, trauma, oral behaviors, as well as psychological distress [1, 5]. Due to their multifactorial etiology, treatment approaches for TMDs can vary widely, from non-invasive interventions such as patient education/self-management, psychological, pharmacological, physical, and appliance therapy, to invasive procedures including occlusal adjustments/rehabilitation, orthodontics, and closed and open TMJ surgeries [1].

Oral behaviors, spanning both sleep and wakefulness, involve a broad range of activities related to the mouth and jaws, including oral parafunction which refers specifically to activities beyond physiological functioning. Consequently, oral behaviors can be classified into sleeping-state (SSA), waking-state non-functional (WNA), and waking-state functional (WFA) oral activities using the Oral Behavior Checklist (OBC) of the DC/TMD [7, 8]. SSAs involve teeth clenching/grinding and adopting positions that exert pressure on the jaws during sleep. WNAs include teeth clenching/grinding and holding activities, whereas WFAs consist of chewing, talking, singing, and yawning during wakefulness. Though studies have explored the association between oral behaviors and TMD, most have focused on painful TMDs and findings remain equivocal due to variations in case definition methods, particularly for SSAs, and outcome measures [9–12]. Furthermore, specific oral activities associated with different TMD subtypes are not widely investigated, and the connections between oral behaviors, jaw function limitations, and psychological distress in TMD patients are not fully examined [13–15].

The Jaw Functional Limitations Scale (JFLS) is frequently utilized to evaluate the functional status of the masticatory system [16]. It was created to resolve shortcomings in previous measures of jaw limitation, such as insufficient item definitions, overlap in items between functional and psychosocial domains, and uncertainty regarding generalizability across orofacial conditions. Both the 8-item (JFLS-8) and 20-item (JFLS-20) iterations of the JFLS exhibit good psychometric properties and are integral components of the DC/TMD [2, 16–18]. The JFLS-20 assesses overall jaw function and includes subscales for mastication, jaw mobility, and emotional/verbal expression [16]. Painful TMD, pain catastrophizing, oral behaviors (especially sleep/awake bruxism), as well as disturbed sleep have been associated with heightened limitations in jaw function, and these relationships may be partial to psychological distress [13, 14, 19–22].

Considering the above, this study aimed to accomplish three primary objectives: (i) determine the frequency of specific oral behaviors associated with various TMD subtypes, (ii) compare the extent of oral behaviors and limitations in jaw function, alongside levels of psychological distress among different TMD patient groups, and (iii) identify correlations between oral behaviors, jaw functional limitation, and psychological distress. The research hypotheses were as follows: (i) the frequency of specific oral behaviors varied among the three TMD subtypes, (ii) individuals with painful TMDs had more jaw overuse behaviors, experienced greater jaw function impairments, and reported higher levels of psychological distress compared to those with non-painful intra-articular conditions, and (iii) there was a moderate correlation between oral behaviors and both jaw functional limitations and psychological distress in TMD patients.

## Materials and methods

### Study population

This retrospective study received approval from the Institutional Review Board of Seoul National University Dental Hospital (ERI22001) and was exempted from the necessity of obtaining informed consent. Nevertheless, all patients were made aware and agreed to the utilization of their deidentified medical records for potential teaching and research purposes upon their registration with the hospital. Anonymized data which was part of a large-scale collaborative study concerning the phenotyping of East Asian TMD patients were obtained from consecutive "initial-visit" patients seeking TMD treatment at a university-affiliated oral medicine clinic in South Korea from January 2020 to December 2021 during routine diagnostic procedures. Utilizing the G*Power software (version 3.1.9.3) and Analysis of Variance (ANOVA) model, a minimum of 390 patients were required to achieve a power of 95% and an alpha error of 0.05. This was under the assumption of a small effect size of 0.2 when comparing OBC scores across three groups [7, 23]. To qualify for the study, patients must be 19 years old or older, fluent in Korean, and experiencing pain in the jaw, temple, or front of the ear, jaw joint sounds, and/or jaw locking. Exclusion criteria include prior orofacial trauma, non-TMD pain, debilitating physical or mental impairments, and incomplete questionnaires. During the initial appointment, patients provided demographic information and completed various questionnaires, including the official Korean DC/TMD Symptom Questionnaire (SQ), OBC, JFLS-20, Patient Health Questionnaire-9 (PHQ-9), and General Anxiety Disorder Scale-7 (GAD-7) [2, 8, 16, 24, 25].

### Categorization of TMD patients

The DC/TMD SQ comprises fourteen items evaluating the five major TMD symptoms, specifically TMD/orofacial pain, headache, Temporomandibular joint (TMJ) noises, closed, and open locking, over the past 30 days. Upon completion of the SQ and other questionnaires, patients underwent protocolized clinical examinations conducted by two Korean national board-certified TMD and orofacial pain specialists who were trained and calibrated in the DC/TMD. The examination assessed pain locations, pain on palpation and jaw movement, jaw movements and deviations, as well as TMJ sounds. All patients received cone-beam computed tomography (CBCT) to detect osseous changes in the TMJs. Magnetic resonance imaging (MRI) was specifically indicated for individuals experiencing protracted restricted mouth opening or suspected TMJ tumors. DC/TMD diagnoses were then rendered based on the patient’s symptoms, clinical signs, and accompanying diagnostic imaging utilizing the diagnostic algorithms of the DC/TMD. Patients were grouped into PT, IT, and CT categories for statistical analysis [6].

### Study measures

#### OBC

The OBC consists of twenty-one items evaluating the frequency of sleeping-state and waking-state oral behaviors. Items are appraised using a 5-point response scale, spanning from "none of the time" with 0 points to "4–7 nights per week" or "all of the time" with 4 points. Total OBC scores, calculated by summing the scores for all 21 items, indicate the overall extent of jaw overuse behaviors and are classified into normal (0 to 16 points), low (17 to 24 points), and high (25 to 84 points) categories [8, 12]. SSA subscale scores are derived from summing items 1 and 2. Similarly, WNA and WFA subscale scores are calculated by summing items 3–7, 11 and 12–13, 17–20 respectively, as specified by Donnarumma et al. [7]. Higher SSA, WNA, and WFA scores indicate heightened levels of oral activities during sleep and wakefulness.

#### JFLS-20

The JFLS-20 comprises twenty items, evaluating a global scale (JFLS-8) for jaw functional status, along with three subscales for mastication, vertical jaw mobility, and verbal/non-verbal communication [16, 26]. Items are appraised with an eleven-point numerical scale, where "0" denotes "no limitation," and "10" corresponds to "extreme limitation". The global scale and three subscale scores are calculated based on the designated items, with higher scores indicating increased levels of "jaw malfunction" or "jaw function disability" [26].

#### PHQ-9 and GAD-7

Depressed and anxious moods were evaluated using the PHQ-9 and GAD-7 which consist of 9 and seven items accordingly [24, 25, 27]. Items are appraised using a 4-point response scale, varying from “not at all’ with 0 points to “nearly every day” with 3 points. Total PHQ-9 and GAD-7 scores, ranging from 0–27 and 0–21 points respectively, are computed by summing the scores across all 9 and 7 items. While depression is categorized as mild (≥ 5 points), moderate (≥ 10 points), moderately severe (≥ 15 points), and severe (≥ 20 points), anxiety is classified into mild (≥ 5 points), moderate (≥ 10 points), and severe (≥ 15 points) categories correspondingly.

### Statistical analyses

Statistical analyses were performed using the IBM SPSS statistical software (version 26.0, IBM Corporation, Armonk, New York, USA) at a significance level of 0.05. Quantitative data were reported as frequencies with percentages and evaluated with Chi-square and post-hoc Z tests. Quantitative data were expressed as means with standard deviations (SDs) and medians with interquartile ranges (IQRs). Normality testing using Shapiro–Wilk’s test was conducted, revealing a non-normal distribution. Consequently, Kruskal–Wallis and post-hoc Mann–Whitney U tests with Bonferroni correction, along with Spearman’s rank-order correlation and logistic regression analysis, were employed for quantitative data. Correlation coefficients between variables (*r*_*s*_) were classified into weak (≥ 0.1), moderate (≥ 0.4), strong (≥ 0.7), and very strong (≥ 0.9) categories [28]. To identify the biopsychosocial and behavioral predictors for PT, IT, and CT, univariate and multivariate logistic regression analyses were carried out. A stepwise variable selection technique with a threshold of p < 0.10 was applied to eliminate insignificant factors. Results were presented as odds ratios (ORs) with 95% confidence intervals (95% CI).

## Results

Of the 1005 anonymized patient records scrutinized, 305 were excluded due to either missing information or incomplete questionnaires. The final study sample consisted of 700 patients, with a mean age of 37.4 ± 15.7 years, of whom 70.3% were female. Among them, 12.6%, 15.1%, and 72.3% received diagnoses of PT, IT, and CT, respectively. Significant differences in sex distribution (females – CT > PT; males – PT > CT), age (PT > CT, IT), and TMD duration (CT > PT, IT) were observed within the TMD groups (Table 1).

**Table 1:** Demographic characteristics of the TMD patients

Variable	All patients	Pain-related TMDs [PT]	Intra-articular TMDs [IT]	Combined TMDs [CT]	P-value *Post-hoc*
Total					
n (%)	700 (100)	88 (12.6)	106 (15.1)	506 (72.3)	
Sex					**0.015**
Females, n (%)	492 (70.3)	53 (60.2)	68 (64.2)	371 (73.3)	CT > PT
Males, n (%)	208 (29.7)	35 (39.8)	38 (35.8)	135 (26.7)	PT > CT
Age					
Mean (SD)	37.37 (15.65)	41.49 (16.05)	34.85 (15.31)	37.18 (15.54)	**0.005**
Median (IQR)	32.00 (24.00)	38.50 (28.50)	29.00 (17.25)	32.00 (24.00)	PT > CT,IT
TMD duration					
Mean (SD)	35.94 (65.88)	23.07 (46.87)	25.29 (52.80)	40.41 (70.55)	** < 0.001**
Median (IQR)	9.00 (35.00)	4.00 (23.00)	2.00 (24.00)	12.00 (46.00)	CT > PT,IT

SD: standard deviation; IQR: Interquartile range. Results of *Kruskal–Wallis/Mann–Whitney U tests and ^Chi-square/Z tests. Bold indicates p < *0.05*

Table 2 shows the distribution of the various sleeping-state and waking-state oral behaviors among the PT, IT, and CT groups. For all TMD subtypes, the two most prevalent oral activities were item 1 (clench or grind teeth when asleep) and item 2 (sleep in a position that puts pressure on the jaw), which ranged from 32.1 to 44.3%. This was followed by item 16 (chew food on one side) and item 21 (hold telephone between your head and shoulders), which varied from 22.5 to 27.3%. Except for item 15 (lean with hand on the jaw), no significant differences were noted in the frequencies of various oral activities among the TMD groups.

**Table 2:** Distribution of the various sleeping-state and waking-state oral behaviors for the three TMD subtypes

Items	All patients n (%)	Pain-related TMD [PT] n (%)	Intra-articular TMD [IT] n (%)	Combined TMD [CT] n (%)	P-value
Sleeping-state oral activities					
I1. Clench or grind teeth when asleep	248 (35.4)	39 (44.3)	34 (32.1)	175 (34.6)	0.156
I2. Sleep in a position that puts pressure on the jaw	290 (41.4)	39 (44.3)	38 (35.8)	213 (42.1)	0.416
Waking-state oral activities					
I3. Grind teeth^	2 (0.3)	0 (0.0)	0 (0.0)	2 (0.4)	1.000
I4. Clench teeth	42 (6.0)	3 (3.4)	6 (5.7)	33 (6.5)	0.519
I5. Press, touch, or hold teeth together other than while eating	92 (13.1)	14 (15.9)	11 (10.4)	67 (13.2)	0.521
I6. Hold, tighten, or tense muscles without clenching or bringing teeth together	81 (11.6)	11 (12.5)	12 (11.3)	58 (11.5)	0.958
I7. Hold or jut jaw forward or to the side^	26 (3.7)	5 (5.7)	3 (2.8)	18 (3.6)	0.544
I8. Press tongue forcibly against teeth^	28 (4.0)	6 (6.8)	5 (4.7)	17 (3.4)	0.226
I9. Place tongue between teeth	44 (6.3)	6 (6.8)	10 (9.4)	28 (5.5)	0.315
I10. Bite, chew, or play with your tongue, cheeks, or lips^	24 (3.4)	2 (2.3)	3 (2.8)	19 (3.8)	0.842
I11. Hold jaw in rigid or tense position	34 (4.9)	2 (2.3)	3 (2.8)	29 (5.7)	0.218
I12. Bite or hold objects between the teeth^	14 (2.0)	0 (0.0)	2 (1.9)	12 (2.4.)	0.453
I13. Use chewing gum^	7 (1.0)	1 (1.1)	1 (0.9)	5 (1.0)	1.000
I14. Play musical instruments that involve use of mouth or jaw^	1 (0.1)	1 (1.1)	0 (0.0)	0 (0.0)	0.126
I15. Lean with hand on the jaw	40 (5.7)	5 (5.7)	0 (0.0)	36 (6.9)	**0.020**
I16. Chew food on one side	165 (23.6)	24 (27.3)	27 (25.5)	114 (22.5)	0.553
I17. Eat between meals	102 (14.6)	16 (18.2)	9 (8.5)	77 (15.2)	0.120
I18. Sustained talking	96 (13.7)	11 (12.5)	14 (13.2)	71 (14.0)	0.916
I19. Singing^	15 (2.1)	0 (0.0)	3 (2.8)	12 (2.4)	0.318
I20 Yawning	98 (14.0)	14 (15.9)	8 (7.5)	76 (15.0)	0.113
I21. Hold telephone between your head and shoulders	165 (23.6)	24 (27.3)	27 (25.5)	114 (22.5)	0.553

*Results of Chi-square test/Z tests. Bold indicates p* < *0.05*

Table 3 presents the mean/median scores for OBC, JFLS-20, PHQ-9, and GAD-7 across the different TMD subtypes. While total OBC, SSA, WNA, and WFA subscale scores were statistically insignificant, considerable variations in the occurrence of low (IT > PT, CT) and high (PT, CT > IT) jaw overuse behaviors were discerned. For jaw functional status, significant differences in global, mastication, mobility, and communication subscale scores (PT, CT > IT) were observed. Additionally, significant differences in depression (PT, CT > IT) and anxiety (CT > IT) were perceived.

**Table 3:** Mean/median oral behavior, jaw functional limitation, and psychological distress scores for the three TMD subtypes

Variable	All patients	Pain-related TMD [PT]	Intrai-articular TMD [IT]	Combined TMD [CT]	P-value *Post-hoc*
*Oral behaviors*					
Total OBC score					
Mean (SD)	15.63 (9.34)	16.15 (9.12)	14.19 (7.64)	15.85 (9.69)	0.412
Median (IQR)	14.00 (12.00)	13.50 (11.75)	14.00 (9.25)	15.00 (12.00)	
Severity					**0.030**
No, n (%)	11 (1.6)	0 (0.0)	2 (1.9)	9 (1.8)	
Low, n (%)	583 (83.3)	71 (80.7)	98 (92.5)	414 (81.8)	IT > PT,CT
High, n (%)	106 (15.1)	17 (19.3)	6 (5.7)	83 (16.4)	PT,CT > IT
Sleeping-state (SSA) OA score					
Mean (SD)	3.54 (2.48)	3.78 (2.51)	3.23 (2.36)	3.57 (2.50)	0.310
Median (IQR)	4.00 (3.00)	4.00 (3.00)	3.00 (4.00)	4.00 (3.00)	
Waking-state non-functional OA (WNA) score					
Mean (SD)	3.71 (3.63)	4.00 (3.62)	3.06 (3.14)	3.79 (3.72)	0.154
Median (IQR)	3.00 (5.00)	3.00 (5.00)	2.00 (3.25)	3.00 (5.00)	
Waking-state functional OA (WFA) score					
Mean (SD)	4.68 (3.16)	4.52 (2.84)	4.33 (2.74)	4.78 (3.29)	0.564
Median (IQR)	4.00 (4.00)	4.00 (4.00)	4.00 (4.00)	4.00 (4.00)	
*Jaw functional limitations*					
Global score					
Mean (SD)	2.18 (1.83)	2.49 (2.01)	0.98 (1.18)	2.37 (1.82)	< 0.001*
Median (IQR)	1.87 (2.66)	2.13 (3.14)	0.51 (1.61)	2.18 (2.55)	PT,CT > IT
Mastication					
Mean (SD)	2.77 (2.29)	3.22 (2.51)	1.36 (1.72)	2.98 (2.25)	< 0.001*
Median (IQR)	2.58 (3.66)	3.08 (4.67)	0.75 (2.21)	3.00 (3.50)	PT,CT > IT
Mobility					
Mean (SD)	2.76 (2.25)	2.92 (2.35)	1.26 (1.63)	3.05 (2.23)	< 0.001*
Median (IQR)	2.5 (3.75)	2.50 (4.13)	0.75 (2.00)	3.00 (3.28)	PT,CT > IT
Communication					
Mean (SD)	1.00 (1.71)	1.32 (2.08)	0.33 (0.82)	1.09 (1.75)	< 0.001*
Median (IQR)	0.20 (1.25)	0.25 (1.45)	0.00 (0.25)	0.25 (1.50)	PT,CT > IT
*Psychological distress*					
Depression (PHQ-9) score					
Mean (SD)	4.68 (5.13)	5.33 (5.19)	3.39 (4.03)	4.84 (5.28)	0.012*
Median (IQR)	3.00 (5.00)	4.00 (7.75)	2.00 (5.00)	3.00 (5.00)	PT,CT > IT
Anxiety (GAD-7) score					
Mean (SD)	4.01 (4.59)	4.01 (4.08)	2.97 (3.77)	4.22 (4.80)	0.041*
Median (IQR)	2.00 (4.00_	3.00 (7.00)	2.00 (5.00)	3.00 (5.00)	CT > IT

SD: standard deviation; IQR: Interquartile range; OA: oral activity. Results of *Kruskal–Wallis/Mann–Whitney U tests and ^Chi-square test/Z tests. Bold indicates p < *0.05*

Tables 4 and 5 display the results of correlation and logistic regression analyses. Total OBC scores displayed a strong correlation with WNA scores (*r*_*s*_ = 0.79), moderately strong associations with both SSA and WFA scores (*r*_*s*_ = 0.64–0.68), and near-moderate relationships with depression (*r*_*s*_ = 0.37) and anxiety (*r*_*s*_ = 0.37). Additionally, SSA and WNA were moderately correlated (*r*_*s*_ = 0.50), while near-moderate relationships were observed between WNA and both depression (*r*_*s*_ = 0.39) and anxiety (*r*_*s*_ = 0.36). A moderately strong association was perceived between depression and anxiety (*r*_*s*_ = 0.69). Although univariate analysis suggested links between different TMD subtypes and various factors, multivariate analysis revealed that PT was influenced by sex (OR = 0.55; 95% CI = 0.34–0.88) and age (OR = 1.02; 95% CI = 1.01–1.04), whereas IT was associated with jaw functional limitation (OR = 0.52; 95% CI = 0.42–0.63). Furthermore, the odds of CT were increased by sex (OR = 1.59; 95% CI = 1.10–2.29) and jaw functional limitation (OR = 1.27; 95% CI = 1.14–1.41).

**Table 4:** Correlations between TMD duration, oral behaviors, jaw functional limitation, depression, and anxiety scores

Variable	TD	TO	SSA	WNA	WFA	FL	PD
TD	-	-	-	-	-	-	-
TO	0.24***	-	-	-	-	-	-
SSA	0.19***	0.68***	-	-	-	-	-
WNA	0.21***	0.79***	0.50***	-	-	-	-
WFA	0.14***	0.64***	023***	0.28***	-	-	-
FL	0.07	0.12**	0.01	0.11**	0.15***	-	-
PD	0.19***	0.37***	0.23***	0.39***	0.21***	0.16***	-
PA	0.15***	0.37***	0.24***	0.36***	0.24***	0.18***	0.69***

*TD: TMD duration; TO: Total oral behavior (OBC) score; SSA: Sleeping-state oral activity score; WNA: Waking-state non-functional oral activity scores; WFA: Waking-state functional oral activity score; FL: global jaw functional limitation (JFLS-20) scale score; PD* = depression (PHQ-9) score; PA: anxiety (GAD-7) score. Results of Spearman’s correlation. *indicates p < 0.05, ** indicates p < 0.01, *** indicates p < *0.001, and bold specifies correlation coefficient* > *0.4*

**Table 5:** Predictors for pain-related, intra-articular, and combined TMDs

	Univariate		Multivariate	
Variables	Odds ratio (95% CI)	P-value*	Odds ratio (95% CI)	P-value
*Pain-related TMD*				
Sex				
Male	Reference		Reference	
Female	0.60 (0.38 – 0.95)	0.028*	0.55 (0.34 – 0.88)	**0.013**
Age	1.02 (1.00 – 1.03)	0.009*	1.02 (1.01 – 1.04)	**0.003**
Oral behaviors				
All OA (total OBC)	1.01 (0.98 – 1.03)	0.581		
Sleeping-state OA	1.05 (0.96 – 1.15)	0.327		
Waking-state non-functional OA	1.02 (0.97 – 1.09)	0.428		
Waking-state functional OA	0.98 (0.91 – 1.06)	0.612		
Jaw functional limitations (JFLS-20)	1.11 (0.99 – 1.24)	0.088		
Depression (PHQ-9)	1.03 (0.99 – 1.07)	0.206		
Anxiety (GAD-7)	1.00 (0.95 – 1.05)	0.997		
*Intra-articular TMD*				
Sex				
Male	Reference			
Female	0.72 (0.46 – 1.11)	0.135		
Age	0.99 (0.97 – 1.00)	0.073		
Oral behaviors				
All OA (total OBC)	0.98 (0.96 – 1.00)	0.084		
Sleeping-state OA	0.94 (0.87 – 1.02)	0.157		
Waking-state non-functional OA	0.94 (0.88 – 1.00)	0.046*		
Waking-state functional OA	0.96 (0.89 – 1.03)	0.220		
Jaw functional limitations (JFLS-20)	0.51 (0.42 – 0.61)	0.001 < *	0.52 (0.42 – 0.63)	** < 0.001**
Depression (PHQ-9)	0.93 (0.88 – 0.98)	0.005*		
Anxiety (GAD-7)	0.93 (0.88 – 0.99)	0.013*		
*Combined TMD*				
Sex				
Male	Reference		Reference	
Female	1.66 (1.17 – 2.36)	0.005*	1.59 (1.10 – 2.29)	**0.014**
Age	1.00 (0.99 – 1.01)	0.604		
Oral behaviors				
All OA (total OBC)	1.01 (0.99 – 1.03)	0.330		
Sleeping-state OA	1.01 (0.95 – 1.08)	0.682		
Waking-state non-functional OA	1.03 (0.98 – 1.07)	0.310		
Waking-state functional OA	1.04 (0.98 – 1.10)	0.175		
Jaw functional limitations (JFLS-20)	1.27 (1.15 – 1.41)	0.001 < *	1.27 (1.14 – 1.41)	0.001 < *
Depression (PHQ-9)	1.02 (0.99 – 1.06)	0.187		
Anxiety (GAD-7)	1.04 (1.00 – 1.08)	0.044*		

*OA: oral activities. Results of univariate and multivariate logistic regression analyses. Bold indicates p* < *0.05*

## Discussion

This study examined oral behaviors in various TMD subtypes, assessing their frequency, extent, and associations with jaw functional limitation, depression, and anxiety. The second research hypothesis, indicating that individuals with painful TMD displayed more pronounced limitations in jaw function and elevated psychological distress when compared to those with non-painful intra-articular conditions, was confirmed. However, the first and third hypotheses were not supported, as there were no substantial differences in the prevalence of specific sleeping-state/waking-state oral behaviors among TMD subtypes, and the correlations between oral behaviors and both jaw functional limitation and psychological distress were generally weak. All psychosocial and behavioral measures employed have sound psychometric properties and are integral components of Axis II of the DC/TMD [2, 8, 16, 24, 25]. Used in conjunction with Axis I of the DC/TMD and its tiered reporting framework, the standardized approach offers an evidence-based biopsychosocial assessment methodology, thereby facilitating future comparisons across diverse cultural and geographical settings [2, 6]. The PT group had a higher percentage of males compared to the CT group, and contrariwise, the CT group had a higher proportion of females than the PT group. Moreover, the PT group consisted of older individuals, whilst the CT group had a more prolonged history of TMD contrasted to the other groups. The findings corroborated previous studies, demonstrating the contributory roles of sex and age in the pathophysiology of TMD [4, 29–31]. The extended TMD duration observed in the CT group could be attributed to the time taken to develop both TMD pain and dysfunction, prompting individuals to seek treatment for their symptoms.

### Oral behaviors among TMD subtypes

Oral behaviors have been recognized as contributing factors to both pain-related and intra-articular TMDs [1, 9, 10, 12, 14, 29]. Regardless of TMD subtypes, the prevalence of sleep-related oral activities, specifically sleeping in a “jaw-compressing” position (41.4%) and teeth clenching or grinding (35.4%), was higher than for waking-state activities (0.1 to 23.6%), even though both sleep-related activities occur during an unconscious state [32]. Unilateral chewing (23.6%) and cradling a telephone (23.6%) were the most frequently reported waking-state oral activities. The findings differed somewhat from those of Xu et al., who identified the four most prevalent oral behaviors among Chinese TMD patients as sleeping in a “jaw-compressing” position (52.9%), unilateral chewing (47.5%), yawning (38.4%), and eating between meals (36.3%) [13]. Despite the differences, East Asian TMD patients share two common oral behaviors, namely sleeping in a “jaw-compressing” position and unilateral chewing. The former includes sleeping on the stomach or side, pushing the jaw backward or to the side, as well as turning the head to the side when sleeping. These sleeping positions may heighten craniocervical-mandibular tension, potentially exacerbating TMD signs/symptoms. Unilateral chewing, or chewing-side preference, has been associated with shorter and more displaced condyles, a steeper articular eminence, alongside a deeper glenoid fossa, and linked to a higher prevalence of TMD signs/symptoms [33]. While there were no significant differences in total OBC, SSA, WNA, and WFA subscale scores among the three TMD subtypes, patients with painful TMD exhibited a three-fold greater occurrence of “high” jaw overuse behaviors. Findings contrast with those of Donnarumma et al., who determined that WNA and WFA were differentially associated with painful and non-painful TMD conditions [7]. Given the methodological similarities, the discrepancies could be explained partly by genetic and environmental distinctions between East-Asian and Western TMD patients [34].

### Jaw function status and psychological distress among TMD subtypes

Unlike the OBC scores, substantial differences in global and subscale JFLS scores were noted between patients with painful TMD, namely PT and CT, and those with non-painful IT. Hence, TMD pain is associated with increased overall limitations in jaw function, as well as difficulties in chewing/biting, opening/closing the mouth, and expressing emotions/speaking. Findings align with that of Fetai et al., demonstrating that jaw functional limitation is influenced more by pain-related than intra-articular conditions. Additionally, pain intensity was found to exert a greater impact than pain chronicity [19]. The role of pain catastrophizing, an exaggerated negative mental response to pain, could be crucial, as it has been related to both pain intensity and jaw functional limitation [20, 35].

Substantial variations in psychological distress were also apparent within the various TMD subtypes. Patients diagnosed with PT and CT exhibited significantly higher depression levels than those with IT, while individuals with CT demonstrated notably elevated anxiety levels compared to those with IT. In their systematic review, Reis et al. presented similar observations, concluding that patients with TMD pain experience higher levels of depression and anxiety compared to those with non-painful TMD. This phenomenon was also evident in non-clinical populations affected by TMD [37]. The complex relationship between pain and psychological distress, where the two issues coexist and reinforce each other, is increasingly recognized. This interaction may be partially mediated by shared genetic susceptibility and neural mechanisms [38, 39].

### Correlation and logistic regression analyses

Total OBC scores showed robust correlations with SSA, WNA, and WFA scores, endorsing the new OBC grouping and scoring method presented by Donnarumma et al. [7]. SSA and WNA were moderately correlated, suggesting a plausible link between sleep and awake bruxism. This affiliation may be explained partly by common risk factors, particularly psychological distress [40]. Overall jaw overuse behaviors (total OBC) and WNA exhibited near-moderate relationships with both depression and anxiety. The findings corroborated prior studies specifying positive connections between oral parafunction and psychological distress [11, 13]. However, the correlation between oral behaviors and jaw functional limitation was found to be weak or very weak, deviating from the results of other studies [13, 14]. This incongruity can be attributed to divergent pain characteristics accompanying different oral behaviors, consequently impacting jaw function [14, 19]. While evidence supporting the causal relationship between SSA and TMD remains inconclusive, TMD pain has recently been directly linked to WNA, specifically awake bruxism, stress, and TMJ dysfunction [10, 11, 41]. The robust association between depression and anxiety observed was also reported in other East Asian TMD patients [42]. Their co-morbidity has been attributed to stress-inducing life events, common negative emotional effects, dysfunctional cognitive processes, and mutual genetic or biological vulnerabilities [43].

Multivariate analysis indicated that, unlike variables such as sex, age, and jaw functional status, oral behaviors do not predict the various TMD subtypes. More explicitly, the odds of PT were 45% lower in females, whereas an increase in age was associated with a 2% higher likelihood of PT. Furthermore, an inverse relationship was noted between jaw functional limitation and IT, indicating a 48% decrease in the odds of limitations in jaw function for patients with IT. The likelihood of CT was increased by 59% in females and by 27% in the presence of jaw functional limitation. Gender differences in TMD prevalence are well established, but variations in sex-specific TMD subtypes are not widely reported, partly due to the lack of a structured reporting framework [1, 4, 5]. Under a level 2 reporting, which categorizes DC/TMD Axis I subtypes into PT, IT, and CT without overlapping principal diagnoses, male TMD patients appear to exhibit a greater predisposition to PT, while female patients seem more susceptible to a combination of PT and IT. Though the exact reasons for this observation are unknown, TMDs may be more closely linked to physical factors in males and to chronic pain, along with hormonal fluctuations in females. This connection renders females more vulnerable to both pain-related and intra-articular conditions, including TMJ disc displacements and degeneration [44, 45]. In addition to physiological alterations, age-related experiential changes, such as workplace and financial stress, as well as domestic and health issues, may contribute to an increased likelihood of TMD pain with advancing age [30, 44–46]. Furthermore, older adults might exhibit heightened sensitivity to mechanically evoked pain compared to their younger counterparts [47]. The associations between jaw functional limitation and both IT and CT may be linked to pain-related jaw disability [19. 35]. Intra-articular conditions, by themselves, are devoid of pain and do not usually adversely affect jaw function. Conversely, limitations in jaw function are linked to TMD-related pain intensity, chronicity, and catastrophization [15, 19, 20, 35]. Even so, recent findings indicate that jaw functional limitation is not correlated with somatization and psychological distress [48].

### Study limitations

Despite employing the evidenced-based DC/TMD methodology, this observational study still has inherent limitations. First, the research focused solely on Korean TMD patients and requires validation in other countries and among different racial groups before the findings can be generalized. Second, the cross-sectional study design employed precluded the determination of causal relationships between oral behaviors, jaw functional status, and psychological distress. Although prospective cohort and nested case–control studies provide the potential to establish causation, the presence of confounding variables, such as comorbid medical conditions or other behavioral factors, and bias can influence the results, making it challenging to confirm definitive associations. Third, the DC/TMD Axis II measures utilized, namely the OBC, JFLS, PHQ-9, and GAD-7, relied on patient self-reporting, introducing potential information biases such as recall bias, social desirability, and other partialities [49]. Lastly, the study explored the frequency of oral behaviors but did not delve into pain characteristics that can affect jaw function and contribute to psychological distress [19, 50].

## Conclusion

In the TMD patients evaluated, 12.6% were diagnosed with PT, 15.1% with IT, and the majority, 72.3%, with CT. For all TMD subtypes, sleep-related oral activities, specifically sleeping in a “jaw-compressing” position (41.4%) and teeth clenching or grinding (35.4%), were more prevalent than waking-state activities (0.1 to 23.6%). While no substantial differences in overall jaw overuse behavior, sleeping-state, waking-state non-functional, and functional oral activities were observed among the TMD groups, patients with painful TMD (PT and CT groups) exhibited significantly greater limitations in jaw function and higher levels of depression. Furthermore, individuals with CT also had higher anxiety levels compared to those with IT. Overall jaw overuse behaviors showed robust correlations to both waking-state non-functional and functional oral activities endorsing the new OBC grouping and scoring method. Additionally, overall jaw use behaviors and waking-state non-functional activities demonstrated near-moderate associations with depression and anxiety, affirming the positive connections between oral parafunction and psychological distress. However, the relationship between oral behaviors and jaw functional limitation was found to be weak. Multivariate analysis indicated that sex, age, and jaw functional status, but not oral behaviors, predicted the various TMD subtypes. Male and older TMD patients appear to have a greater predisposition to pain-related conditions, while female patients seem more susceptible to a combination of pain-related and intra-articular problems. The occurrence of jaw functional limitation, often linked to TMD pain, increased the risk of combined TMD. Collectively the findings suggest that oral behaviors, by themselves, are not associated with pain-related and intra-articular TMD. Future research should prioritize elucidating the intricate mechanisms underlying the relationship between specific oral behaviors and TMD pain characteristics while considering the influence of psychological distress and other psychosocial factors.

## Data Availability

The datasets generated and analysed during the current study are not publicly available due to ethical reasons but are available from the corresponding author on reasonable request.

## Abbreviations

CT: Combined Temporomandibular disorder/s
DC/TMD: Diagnostic criteria for Temporomandibular disorders
GAD-7: General Anxiety Disorder Scale-7
IT: Intra-articular Temporomandibular disorder/s
JFLS-20: Jaw Functional Limitations Scale-20
OBC: Oral Behavior Checklist
PHQ-9: Patient Health Questionnaire-9
PT: Pain-related Temporomandibular disorder/s
SQ: Symptom questionnaire
SSA: Sleeping-state oral activities
WFA: Waking-state functional oral activities
WNA: Waking-state non-functional oral activities
